# An Information-Theoretic Proof of a Hypercontractive Inequality

**DOI:** 10.3390/e26110966

**Published:** 2024-11-11

**Authors:** Ehud Friedgut

**Affiliations:** Faculty of Math and Computer Science, Weizmann Institute of Science, Rehovot 7610001, Israel; ehud.friedgut@weizmann.ac.il

**Keywords:** entropy, hypercontractive inequality, boolean functions

## Abstract

The famous hypercontractive estimate discovered independently by Gross, Bonami and Beckner has had a great impact on combinatorics and theoretical computer science since it was first used in this setting in a seminal paper by Kahn, Kalai and Linial. The usual proofs of this inequality begin with two-point space, where some elementary calculus is used and then generalised immediately by introducing another dimension using submultiplicativity (Minkowski’s integral inequality). In this paper, we prove this inequality using information theory. We compare the entropy of a pair of correlated vectors in ${\left\{0,1\right\}}^{n}$ to their separate entropies, analysing them bit by bit (not as a figure of speech, but as the bits are revealed) using the chain rule of entropy.

## 1. Introduction

The inequality that we consider in this note is a two-function version of a famous hypercontractive inequality discovered, independently, by Gross [1], Bonami [2] and Beckner [3]. This inequality, first introduced to the combinatorial landscape in a seminal paper [4] by Kahn, Kalai and Linial (henceforth KKL), has become one of the cornerstones of the analytical approach to Boolean functions and theoretical computer science; see, e.g., [5,6,7,8,9,10], and many others. See chapter 16 of [11] for a historical background.

Let ϵ∈(0,1). We will be considering an operator $T_{\epsilon }$, which acts on real-valued functions in ${\left\{0,1\right\}}^{n}$. We consider two equivalent definitions of the operator: a spectral definition and a more probabilistic one. The first definition is defined via the eigenfunctions and eigenvalues of the operator. The eigenfunctions are precisely the Walsh–Fourier variables, $\left\{u_{x}\right\}:x\in {\left\{0,1\right\}}^{n}$, which form a complete orthonormal system under the inner product <f,g>:=E[f(x)g(x)], where the expectation of a function is defined by $E\left[h\left(x\right)\right]=2^{-n}\sum_{x}h\left(x\right)$. We recall the definition of these variables. For $x,y\in {\left\{0,1\right\}}^{n}$,

$$u_{x}\left(y\right)={\left(-1\right)}^{\sum_{i}x_{i}y_{i}}.$$

Given a function $f:{\left\{0,1\right\}}^{n}\to R$ and its unique Fourier expansion, $f=\sum_{x}\hat{f}\left(x\right)u_{x}$, the action of $T_{\epsilon }$ on *f* is defined by

$$T_{\epsilon }\left(f\right)=\sum_{x}\epsilon^{\sum_{i}x_{i}}\hat{f}\left(x\right)u_{x}.$$

This definition of $T_{\epsilon }$ stresses the fact that it “focuses” on the low-frequency part of the Fourier spectrum, an idea that was a crucial element in the KKL proof [4].

For the other definition, let *X* and *Y* be random variables taking values in ${\left\{0,1\right\}}^{n}$. For any distribution of *X*, let *Y* be such that for every coordinate *i*, independently, $Y_{i}$ is chosen so that $Pr\left[X_{i}=Y_{i}\right]=\frac{1+\epsilon }{2}$. (Or, if one prefers the ${\left\{-1,1\right\}}^{n}$ setting, the restriction is $E[X_{i}Y_{i}]=\epsilon$.) Note that if *X* is chosen uniformly, then the marginal distribution of *Y* is also uniform. We call such a pair (X,Y) an ϵ*-correlated pair*. Then, one can define

$$T_{\epsilon }\left(f\right)\left(x\right)=E\left[f\left(Y\right)|X=x\right].$$

It is not hard to verify that these two definitions of $T_{\epsilon }$ are equivalent. The second definition, which is the one we will be working with in this paper, stresses the connection of this operator to random walks and isoperimetric inequalities, as it enables one to bind the probability of an ϵ-correlated pair of random points X,Y to belong to given sets. It also explains the fact (which will be made formal shortly) that $T_{\epsilon }\left(f\right)$ is smoother than *f*, as the operator is an averaging operator. Without further ado, here is the statement of the inequality:

**Theorem** **1** ([1,2,3])**.** *Let $f:{\left\{0,1\right\}}^{n}\to R_{\geq 0}$ and let ϵ∈[0,1]. Then,*

(1) $$|T_{\epsilon }{\left(f\right)|}_{2}\leq {\left|f\right|}_{1+\epsilon^{2}},$$

*where ${\left|g\right|}_{p}:={\left(E[|g\right|}^{p}{])}^{1/p}$ and the expectation is written with respect to a uniform measure.*

**Remarks:**

- There are various refinements of this inequality, either dealing with non-uniform measures, the norm of $T_{\epsilon }$ as an operator ranging from $L^{p}$ to $L^{q}$ for q≠2, products of a base space with more than two points, or a reverse inequality that deals with the case p,q<1; see [10,12,13,14]. It would not be surprising if the method used in this note could be extended to cover such cases too.
- It is not difficult to see that (1) is equivalent to the following: Let $f,g:{\left\{0,1\right\}}^{n}\to R$; let *X* be uniformly distributed on ${\left\{0,1\right\}}^{n}$; and let X,Y be an ϵ-correlated pair. Then,
  (2) $$E\left[f\left(X\right)g\left(Y\right)\right]\leq {\left|f\right|}_{1+\epsilon }{\left|g\right|}_{1+\epsilon }.$$
  This is the inequality proved in this paper.
- A major portion of the hypercontractive inequality’s applications deal with the case where *f* and *g* are Boolean functions. We will start our proof with this setting and then show how a small variation deals with the general case.

In this paper, we apply an information-theoretic approach to proving (2) for Boolean functions, trying to analyse the pair (X,Y) as the coordinates of *X* and *Y* are revealed to us one by one. Since all known direct proofs of (1) use induction, it is not surprising that one should adopt such a sequential approach. The difference is that the usual proofs begin with two-point space and proceed through induction using the submultiplicativity of the product operator and Minkowski’s integral inequality, whereas we use the chain rule of entropy, exposing the bits of the vectors in question one by one and comparing the amount of information about their joint distribution with the information captured by their marginal distributions. Fortunately, it turns out that regardless of the prefixes revealed so far, at every step, the conditional entropies obey the same inequality.

This is not the first application of entropy to this hypercontractive inequality. The connection between hypercontractivity-type inequalities and information theory was perhaps first addressed in [15]. In [16], the dual form of the hypercontractive inequality is proven for the case of comparing the 2-norm and the 4-norm of a low-degree polynomial acting on ${\left\{0,1\right\}}^{n}$. Blais and Tan, [17], managed to improve this approach and, surprisingly, extract the precise optimal hypercontractive constant for comparing the 2-norm and the *q*-norm of such polynomials for all positive even integers *q*. Both these proofs analyse the Fourier space rather than the primal space and use no induction at all.

I will make one final remark regarding the proof in this paper. Although it is not difficult, it is probably, to date, the most involved proof of the inequality from a technical point of view. I believe that it is nonetheless worthwhile to add it to the list of existing proofs because it offers a new point of view which directly addresses the notion of studying the projections of the joint distribution of a pair of ϵ-correlated vectors.

## 2. Main Theorem

### 2.1. The Boolean Case

**Theorem** **2.** *Let ϵ∈[0,1], and let $X,Y\subseteq {\left\{0,1\right\}}^{n}$ be nonempty. Let X be uniformly distributed in ${\left\{0,1\right\}}^{n}$, and let Y be such that for each 1≤i≤n, independently, $Pr\left[X_{i}=Y_{i}\right]=\frac{1+\epsilon }{2}$. Then,*

(3) $$E\left[1_{X}\left(X\right)1_{Y}\left(Y\right)\right]\leq {\left(\mu (X)\mu (Y)\right)}^{\frac{1}{1+\epsilon }}$$

*where μ denotes a uniform measure.*

**Proof.**  For $x,y\in {\left\{0,1\right\}}^{n}$, let a(x,y) denote the number of coordinates on which *x* and *y* agree and d(x,y) be the number of coordinates on which they differ. Then, the theorem is equivalent (by straightforward manipulation) to

(4) $$\log\left(\sum_{x\in X}\sum_{y\in Y}{\left(1+\epsilon \right)}^{a(x,y)}{\left(1-\epsilon \right)}^{d(x,y)}\right)\leq \frac{1}{1+\epsilon }\left(2\epsilon n+\log(|X|)+\log(|Y|)\right),$$

where all logs are base 2. As is usual in proofs using entropy, it suffices, through continuity, to treat the case where ϵ is rational. Let s≤r be natural numbers such that $\frac{1+\epsilon }{2}=\frac{r}{r+s}$ and $\frac{1-\epsilon }{2}=\frac{s}{r+s}$. Then, (2) reduces to

(5) $$\log\left(\sum_{x\in X}\sum_{y\in Y}r^{a(x,y)}s^{d(x,y)}\right)\leq n\left(\log\left(r+s\right)-s/r\right)+\frac{r+s}{2r}\left(\log(|X|\right)+\log(|Y|)).$$

We will express the left-hand side of this expression as the entropy of a random variable and proceed to expand it according to the chain rule. First, let $A_{00},A_{11},A_{10}$ and $A_{01}$ be four disjoint sets with

$$\left|A_{00}\right|=\left|A_{11}\right|=r,\;\left|A_{01}\right|=\left|A_{10}\right|=s.$$

Next, let (X,Y,Z) be a random variable which is distributed uniformly over all triples such that X∈X,Y∈Y, and for every 1≤i≤n, $Z_{i}\in A_{X_{i}Y_{i}}.$ Clearly, *Z* determines *X* and *Y* so that

$$H\left(X,Y,Z\right)=H\left(Z\right)=\log\left(\sum_{x\in X}\sum_{y\in Y}r^{a(x,y)}s^{d(x,y)}\right).$$

Next, for any vector $w\in {\left\{0,1\right\}}^{n}$, we denote $\left(w_{1},\ldots ,w_{i-1}\right):=w_{<i}$. So, from the chain rule, we have

$$H\left(Z\right)=\sum_{i}H\left(Z_{i}|Z_{<i}\right)$$

and

$$H\left(X\right)=\sum_{i}H\left(X_{i}|X_{<i}\right)\leq \log\left(|X|\right),H\left(Y\right)=\sum_{i}H\left(Y_{i}|Y_{<i}\right)\leq \log\left(|Y|\right).$$

Hence, it suffices to prove that

(6) $$H\left(Z\right)\leq \frac{r+s}{2r}\left(H(X)+H(Y)\right)+n\left(\log\left(r+s\right)-s/r\right).$$

By noting that any fixed value of $Z_{<i}$ determines the values of $X_{<i}$ and $Y_{<i}$, we have

$$H\left(X_{i}|Z_{<i}\right)\leq H\left(X_{i}|X_{<i}\right),$$

and

$$H\left(Y_{i}|Z_{<i}\right)\leq H\left(Y_{i}|Y_{<i}\right),$$

so inequality (6) will follow from this. □

**Claim** **1.** *Let $Past=z_{<i}$ be any specific value that $Z_{<i}$ attains. Then,*

$$H\left(Z_{i}|Z_{<i}=Past\right)\leq \frac{r+s}{2r}\left(H\left(X_{i}|Z_{<i}=Past\right)+H\left(Y_{i}|z_{<i}=Past\right)\right)+\log\left(r+s\right)-\frac{s}{r}$$

**Proof.**  We use the condition that $Z_{<i}=Past$, and through manipulating the notation, we drop the dependency in the notation so that, e.g., we write H(X) and H(Z|X) rather than $H(X|Z_{<i}=Past)$ and $H(Z|X,Z_{<i}=Past)$, etc. We also drop the index *_i_* and write *X* for $X_{i}$, etc., so, using the new notation, we want to prove (for all integers r≥s≥0) that

$$H\left(Z\right)\leq \frac{r+s}{2r}\left(H(X)+H(Y)\right)+\log\left(r+s\right)-\frac{s}{r}$$

Note that H(Z)=H(X,Y)+H(Z|X,Y)=H(X,Y)+logr(Pr[X=Y])+logs(Pr[X≠Y]), so we need to prove that

$$\frac{r+s}{2r}\left(H(X)+H(Y)\right)-H\left(X,Y\right)-\log r\left(Pr\left[X=Y\right]\right)-\log s\left(Pr\left[X\neq Y\right]\right)+\log\left(r+s\right)-\frac{s}{r}\geq 0$$

Since this expression is invariant when *r* and *s* are multiplied by any positive constant, we can set r=1 and denote δ:=s/r. Next, for a joint distribution of *X* and *Y* over ${\left\{0,1\right\}}^{2}$ and $\left(i,j\right)\in {\left\{0,1\right\}}^{n}$, let $P_{ij}=Pr\left[X=i,Y=j\right]$. Hence, we want to prove that

(7) $$F_{\delta }\left(\begin{array}{cc} P_{01} & P_{11}\\ P_{00} & P_{10} \end{array}\right):=\frac{1+\delta }{2}\left(H(X)+H(Y)\right)-H\left(X,Y\right)-\log\delta \left(Pr\left[X\neq Y\right]\right)+\log\left(1+\delta \right)-\delta \geq 0$$

We know (and can check directly from the formula) that this equality holds when $X=Y={\left\{0,1\right\}}^{n}$, in which case we have

(8) $$\left(\begin{array}{cc} P_{01} & P_{11}\\ P_{00} & P_{10} \end{array}\right)=\left(\begin{array}{cc} \frac{\delta }{2+2\delta } & \frac{1}{2+2\delta }\\ \frac{1}{2+2\delta } & \frac{\delta }{2+2\delta } \end{array}\right).$$

We wish to show that this is a unique minimum. To simplify the notation (and save using indices), we denote

$$\left(\begin{array}{cc} P_{01} & P_{11}\\ P_{00} & P_{10} \end{array}\right):=\left(\begin{array}{cc} a & b\\ c & d \end{array}\right),$$

and attempt to minimise $F_{\delta }\left(\begin{array}{cc} a & b\\ c & d \end{array}\right)$ under the constraints

a,b,c,d≥0 and a+b+c+d=1.

Using Lagrange multipliers, we deduce (after some simple cancelations) that at a local minimum in the interior of the region in question, one must have

(9) $$\frac{\delta }{a}{\left[\left(a+b)(a+c\right)\right]}^{(1+\delta )/2}=$$

(10) $$\frac{1}{b}{\left[\left(a+b)(b+d\right)\right]}^{(1+\delta )/2}=$$

(11) $$\frac{1}{c}{\left[(a+c)(c+d)\right]}^{(1+\delta )/2}=$$

(12) $$\frac{\delta }{d}{\left[(c+d)(b+d)\right]}^{(1+\delta )/2}.$$

From the fact that (9) · (12) = (10) · (11), we obtain

(13) $$ad=\delta^{2}bc$$

Next, we plug (13) and the restriction a+b+c+d=1 into Equation (9) = (12). This yields

(14) $${\left(\frac{d}{a}\right)}^{(1-\delta )/2}={\left[\frac{1+(\delta^{-2}-1)a}{1+(\delta^{-2}-1)d}\right]}^{(1+\delta )/2}.$$

Note that for every fixed value of b+c, the value of a+d is fixed, so letting *d* grow from 0 to 1−b−c as a=1−b−c−d decreases from 1−b−c to 0, we see that the left-hand side of (14) is increasing and the right-hand side is decreasing. Hence, there exists a single solution, which, by inspection, is a=d. □

We now know that a=d and that $bc=\delta^{-2}a^{2}$ and b+c=1−2a. This shows that *b* and *c* are the roots of the quadratic equation $X^{2}-\left(1-2a\right)X+\delta^{-2}a^{2}=0$. Plugging these roots into Equation (10) = (11) and using a=d yield the following equation for *a*:

(15) $$\frac{\left[1-2a+S\left(a\right)\right]{\left[1-S\left(a\right)\right]}^{1+\delta }}{\left[1-2a-S\left(a\right)\right]{\left[1+S\left(a\right)\right]}^{1+\delta }}=1,$$

where S(a) denotes $\sqrt{1-4a+4\left(1-\delta^{-2}\right)a^{2}}$. Now, *a* can take on values between 0 and 1/2 as long as S(a)≥0, so the relevant range is $0\leq a\leq \frac{\delta }{2+2\delta }$. When $a=\frac{\delta }{2+2\delta }$, as required, S(a)=0 and Equation (15) clearly holds. An elementary calculation shows that for all δ∈[0,1], the left-hand side of (15) is a decreasing function of *a* in the interval $\left[0,\frac{\delta }{2+2\delta }\right]$, so (a,b,c,d) is determined, and there is a unique internal minimum in the region, which we are exploring. (A meticulous reader may check that the derivative of the left-hand side of (15) according to *a* is

$$\frac{16a^{2}\left(1-\delta \right){\left(1-S(a)\right)}^{\delta }{\left(1+S(a)\right)}^{-\delta -2}\left(2a\left(\delta +1\right)-\delta \right)}{\delta^{3}{\left(S(a)+2a-1\right)}^{2}S\left(a\right)},$$

whose sign is determined by 2a(δ+1)−δ, which is negative for all $a\in (0,\frac{\delta }{2+2\delta })$).

What about points on the boundary of the region? We claim that there can be no minima with negative values on the boundary. We deal with two cases: the case of $\left(\begin{array}{cc} a & b\\ c & 0 \end{array}\right)$ with a>0 (and the different rotations of this) and the case of $\left(\begin{array}{cc} 0 & b\\ c & 0 \end{array}\right)$ and its rotation (note that $F_{\delta }\left(\begin{array}{cc} 0 & y\\ z & 0 \end{array}\right)\leq F_{\delta }\left(\begin{array}{cc} y & 0\\ 0 & z \end{array}\right)$).

In the first case, there is a nearby point in the interior of the region, where the value of $F_{\delta }$ is smaller. This is because the derivative of

$$F_{\delta }\left(\begin{array}{cc} a-t & b\\ c & t \end{array}\right)$$

with respect to *t* at t=0 is minus infinity (as one of the summands being derived is tlog(t)). In the second case, the value of $F_{\delta }$ is non-negative,

$$F_{\delta }\left(\begin{array}{cc} 0 & b\\ 1-b & 0 \end{array}\right)=\delta \left(b\log\left(1/b\right)+\left(1-b\right)\log\left(1/\left(1-b\right)\right)\right)+\log\left(1+\delta \right)-\delta ,$$

as $\log_{2}\left(1+\delta \right)\geq \delta$ for all δ∈[0,1].

### 2.2. The General (Non-Boolean) Case

The general case is actually a minor extension of the Boolean one, which follows by adding one more coordinate to each of the random variables *X* and *Y*.

**Theorem** **3.** *Let ϵ∈(0,1), and let $f,g:{\left\{0,1\right\}}^{n}\to R_{\geq 0}$. As before, let X,Y be an ϵ-correlated pair of random variables with X uniformly distributed over ${\left\{0,1\right\}}^{n}$. Then,*

(16) $$E\left[f\left(X\right)g\left(Y\right)\right]\leq {\left|f\right|}_{1+\epsilon }{\left|g\right|}_{1+\epsilon }.$$

**Proof.**  By continuity, it suffices to consider the case where all values of *f* and *g* are rational, and by homogeneity, we can clear common denominators and assume that they are integer-valued. Now, we wish to prove a slight extension of (5), namely

(17) $$\log\left(\sum_{x\in {\left\{0,1\right\}}^{n}}\sum_{y\in {\left\{0,1\right\}}^{n}}r^{a(x,y)}s^{d(x,y)}f\left(x\right)g\left(y\right)\right)\leq$$

(18) $$n\left(\log\left(r+s\right)-s/r\right)+\frac{r+s}{2r}\left(\log\left(\sum_{x\in {\left\{0,1\right\}}^{n}}f{\left(x\right)}^{\frac{2r}{r+s}}\right)+\log\left(\sum_{y\in {\left\{0,1\right\}}^{n}}f{\left(y\right)}^{\frac{2r}{r+s}}\right)\right).$$

To this end, we now define as before the random variables X,Y and *Z*, add two more random integer variables *a* and *b*, and take (X,Y,Z,A,B) uniformly, with (X,Y,Z) defined as before and the additional constraint that A∈{1,…,f(X)} and B∈{1,…,g(Y)}. Now, (17) is precisely $H\left(Z,A,B\right)=H\left(Z\right)+H\left(A|Z\right)+H\left(B|Z\right)=H\left(Z\right)+E_{X}\left[\log\left(f\left(X\right)\right)\right]+E_{Y}\left[\log\left(g\left(Y\right)\right)\right]$. On the other hand, note that for any function *t*, it holds that

$$H\left(X\right)+E_{X}\left[\log\left(t\left(X\right)\right)\right]\leq \log\left(\sum_{x}t\left(x\right)\right).$$

(This is the non-negativity of the Kullbak–Leibler divergence (relative entropy) of *X* and t(X).) In particular,

$$\frac{r+s}{2r}\left(H\left(X\right)\right)+E_{X}\left[\log\left(f\left(X\right)\right)\right]\leq \frac{r+s}{2r}\left(\log\left(\sum_{x\in {\left\{0,1\right\}}^{n}}f{\left(x\right)}^{\frac{2r}{r+s}}\right)\right)$$

and

$$\frac{r+s}{2r}\left(H\left(Y\right)\right)+E_{Y}\left[\log\left(g\left(Y\right)\right)\right]\leq \frac{r+s}{2r}\left(\log\left(\sum_{y\in {\left\{0,1\right\}}^{n}}g{\left(y\right)}^{\frac{2r}{r+s}}\right)\right)$$

To complete the proof of Theorem 3, we just add $E_{X}\left[\log\left(f\left(X\right)\right)\right]+E_{Y}\left[\log\left(g\left(Y\right)\right)\right]$ to both sides of the main inequality that we proved in the Boolean case, namely

$$H\left(Z\right)\leq \frac{r+s}{2r}\left(H(X)+H(Y)\right)+n\left(\log\left(r+s\right)-s/r\right),$$

and we are done. □

## Data Availability

The original contributions presented in the study are included in the article, further inquiries can be directed to the corresponding author.
